# Characterization and bioactivity of novel calcium antagonists - *N*-methoxy-benzyl haloperidol quaternary ammonium salt

**DOI:** 10.18632/oncotarget.6086

**Published:** 2015-10-19

**Authors:** Yi-Cun Chen, Wei Zhu, Shu-Ping Zhong, Fu-Chun Zheng, Fen-Fei Gao, Yan-Mei Zhang, Han Xu, Yan-Shan Zheng, Gang-Gang Shi

**Affiliations:** ^1^ Department of Pharmacology, Shantou University Medical College, Shantou 515041, Guangdong, China; ^2^ Department of Biochemistry and Molecular Biology, Keck School of Medicine, University of Southern California, Los Angeles, California 90033, USA; ^3^ Department of Pharmacy, the First Affiliated Hospital, Shantou University Medical College, Shantou 515041, Guangdong, China; ^4^ Geneheal Biotechnology Co., Ltd, Guangzhou 510000, Guangdong, China; ^5^ Department of Cardiovascular Diseases, the First Affiliated Hospital, Shantou University Medical College, Shantou 515041, Guangdong, China

**Keywords:** calcium, novel calcium antagonists, KCl-induced aortic ring contraction, synthesis

## Abstract

**BACKGROUND AND PURPOSE:**

Calcium antagonists play an important role in clinical practice. However, most of them have serious side effects. We have synthesized a series of novel calcium antagonists, quaternary ammonium salt derivatives of haloperidol with N-p-methoxybenzyl (X_1_), N-m-methoxybenzyl (X_2_) and N-o-methoxybenzyl (X_3_) groups. The objective of this study was to investigate the bioactivity of these novel calcium antagonists, especially the vasodilation activity and cardiac side-effects. The possible working mechanisms of these haloperidol derivatives were also explored.

**EXPERIMENTAL APPROACH:**

Novel calcium antagonists were synthesized by amination. Compounds were screened for their activity of vasodilation on isolated thoracic aortic ring of rats. Their cardiac side effects were explored. The patch-clamp, confocal laser microscopy and the computer-fitting molecular docking experiments were employed to investigate the possible working mechanisms of these calcium antagonists.

**RESULTS:**

The novel calcium antagonists, X_1_, X_2_ and X_3_ showed stronger vasodilation effect and less cardiac side effect than that of classical calcium antagonists. They blocked L-type calcium channels with an potent effect order of X_1_ > X_2_ > X_3_. Consistently, X_1_, X_2_ and X_3_ interacted with different regions of Ca^2+^-CaM-CaV1.2 with an affinity order of X_1_ > X_2_ > X_3_.

**CONCLUSIONS:**

The new halopedidol derivatives X_1_, X_2_ and X_3_ are novel calcium antagonists with stronger vasodilation effect and less cardiac side effect. They could have wide clinical application.

## INTRODUCTION

Calcium antagonists selectively block calcium channel and inhibit the influx of extracellular calcium ions (Ca^2+^). Members of this class are clinically used in the treatment of cardiovascular, cerebrovascular and peripheral vascular spastic diseases [1, 2]. Calcium channel blockers for clinical usage are categorized as dihydropyridines, phenylalkylamine or benzothiazepines. They are not recommended for patients with heart failure, sinus node or atrioventricular node dysfunction due to some unwanted serious side effects [3–5]. This has led to limited or completely restricted use of certain calcium antagonists. Therefore, identifying novel calcium channel blockers with vasodilation activity and less cardiac side effects are needed.

Haloperidol is a classic antipsychotic drug belonging to the butyrophenones group. Our previous study revealed that quaternary ammonium salt derivatives of haloperidol could partly antagonize the contraction of coronary artery and protect myocardial cell against ischemia-reperfusion injury [6, 7]. Subsequent to these findings, a series of derivatives were synthesized, and screened for their vasodilation activity. These haloperidol derivatives were found to have vasodilation effect [8]. It was also noted that some of these derivatives have less cardiac side effects.

In this study, we synthesized three new haloperidol derivatives X_1_, X_2_ and X_3_, with para- meta-, and ortho-substituted *N*-methoxy-benzyl group respectively. We studied their Ca^2+^ antagonistic effect and cardiac side effect in comparison with traditional calcium antagonists. We explored their possible working mechanisms. The results have potential use for developing novel calcium antagonists with stronger activity and less cardiac side effects.

## RESULTS

### Synthesis of para-, meta- and ortho-substituted N-methoxy-benzyl haloperidol derivatives (X_1_, X_2_, X_3_)

**Scheme 1:**
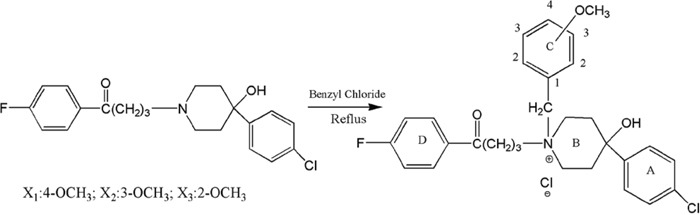
Synthesis of para-, meta- and ortho-substituted N-methoxy-benzyl haloperidol derivatives

The raw material haloperidol was reacted with para-, meta- and ortho-substituted benzyl chloride to form the target compounds X_1_, X_2_ and X_3_ (Scheme 1). These compounds were identified by IR, HNMR and MS (Table S1, S2).

### Effects of haloperidol derivatives on rat hemodynamics

The effect of haloperidol derivatives on heart was investigated. The results showed that the classical calcium antagonists, verapamil and diltiazem at 0.25 mg/kg, 0.5 mg/kg and 1 mg/kg reduced heart rate (HR), left ventricular systolic pressure (LVSP) and ± dp/dt_max_, and simultaneously increased left ventricular end-diastolic pressure (LVEDP), to different levels. Nifedipine lowered LVSP and ± dp/dt_max_ and simultaneously increased LVEDP to different degree. Due to a compensatory reaction, nifedipine slightly increased HR at 0.5 mg/kg and slightly reduced HR at 1 mg/kg. These changes were considered as adverse effects on the heart [16]. However, X_1_, X_2_ and X_3_ had less effect on HR at all concentrations. At concentrations of 0.25 mg/kg and 0.5 mg/kg, X_1_, X_2_ and X_3_ did not affect LVSP, ± dp/dt_max_ and LVEDP. At 1 mg/kg, the X_1_ and X_2_ temporarily reduced the LVSP and +dp/dt_max_, increased LVEDP, but did not affect −dp/dt_max_ (Table 1). In summary, X_1_, X_2_ and X_3_ showed fewer side effects on heart.

**Table 1:** Effects of X series derivatives and classical calcium antagonists on rat hemodynamic

Group	HR	LVSP	LVEDP	+LVdp/dtmax	−LVdp/dtmax
Control	367 ± 52	142.88 ± 15.22	21.31 ± 3.21	1181.82 ± 153.04	966.22 ± 118.22
Ver:0.25 mg/kg	328 ± 28*	132.24 ± 12.74	25.39 ± 7.45	1022.49 ± 130.44	932.24 ± 95.54
Ver: 0.5 mg/kg	302 ± 77**	112.74 ± 21.08**	38.21 ± 8.18**	925.38 ± 172.37*	699.87 ± 91.11**
Ver: 1 mg/kg	298 ± 41**	102.21 ± 14.21**	42.21 ± 6.23**	885.32 ± 122.21*	657.80 ± 85.55**
Nif:0.25 mg/kg	372 ± 29	137.21 ± 18.26	29.33 ± 5.56*	1021.47 ± 132.25	931.56 ± 89.67
Nif: 0.5 mg/kg	397 ± 73*	132.11 ± 11.10*	33.64 ± 8.12*	869.43 ± 136.66*	699.87 ± 68.21**
Nif: 1 mg/kg	311 ± 54*	105.00 ± 12.23**	45.50 ± 6.43**	802.34 ± 110.23*	666.94 ± 83.28**
Dil: 0.25 mg/kg	322 ± 25*	138.54 ± 21.35	30.21 ± 3.99*	1099.22 ± 142.66	787.25 ± 74.25*
Dil: 0.5 mg/kg	321 ± 29*	130.25 ± 17.33*	33.26 ± 4.26*	922.11 ± 129.44*	689.76 ± 77.62**
Dil: 1 mg/kg	304 ± 47**	127.55 ± 21.23**	38.51 ± 7.45**	899.08 ± 128.32*	622.94 ± 84.42**
X_1_: 0.25 mg/kg	355 ± 61	145.32 ± 20.08	20.34 ± 3.06	1043.01 ± 143.24	899.04 ± 142.24
X_1_: 0.5 mg/kg	356 ± 55	145.38 ± 18.06	21.02 ± 3.08	1011.02 ± 152.04	908.21 ± 151.02
X_1_: 1 mg/kg	342 ± 51	132.32 ± 18.66*	29.66 ± 4.33*	946.02 ± 132.23*	884.82 ± 148.09
X_2_: 0.25 mg/kg	365 ± 59	145.98 ± 16.42	22.12 ± 3.76	1029.32 ± 144.08	982.13 ± 154.56
X_2_: 0.5 mg/kg	362 ± 63	146.66 ± 18.04	22.08 ± 3.44	1022.14 ± 142.21	984.12 ± 144.32
X_2_: 1 mg/kg	356 ± 53	138.94 ± 16.06*	26.93 ± 3.26*	902.89 ± 138.88*	955.23 ± 146.08
X_3_: 0.25 mg/kg	358 ± 52	142.94 ± 18.08	23.86 ± 3.65	1129.28 ± 162.26	989.92 ± 161.23
X_3_0.5 mg/kg	362 ± 55	148.98 ± 20.02	24.91 ± 3.07	1221.12 ± 170.02	1021.23 ± 153.77
X_3_1 mg/kg	368 ± 54	152.88 ± 21.04	23.69 ± 3.77	1248.36 ± 182.32	1022.15 ± 162.24

Notes: Ver, verapamil, Nif, nifedipine, Dil, diltiazem.

** P < 0.01;

* P < 0.05 vs Control group.

### Vasodilation on rat aortic rings

**Figure 1:**
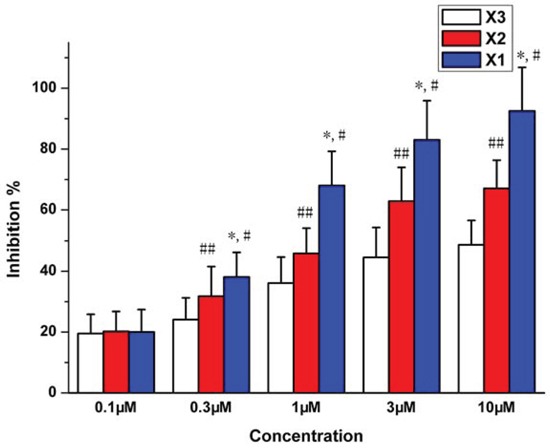
Inhibition of vessel contraction by X_1_, X_2_ and X_3_ on KCl (60 mM)-induced contraction of rat aortic rings *p < 0.05 X_1_ compared with X2, ^#^*p* < 0.05 X1 compared with X_3_, ^##^*p* < 0.05 X2 compared with X3.

The vasodilation activity of X_1_, X_2_ and X_3_ were investigated. The rat model of KCl-induced aortic ring contraction is the classic model *in vitro* for screening the vasodilation drugs [17, 18]. The X_1_, X_2_ and X_3_ at 0.1 to 10 μM showed vasodilation activity to varying degrees in a dose-dependent manner (Figure 1). The IC_50_ of X_1_, X_2_ and X_3_ were 0.432, 4.22 and 7.42 μM (*P* < 0.01), respectively. The maximum inhibition on the vascular ring contraction was 92.5%, 67.08% and 48.66%, respectively for X_1_, X_2_ and X_3_. The order of vasodilation activity was X_1_ > X_2_ > X_3_ (*P* < 0.01).

### Inhibition of KCl-induced rat myocardial extracellular calcium influx

**Figure 2:**
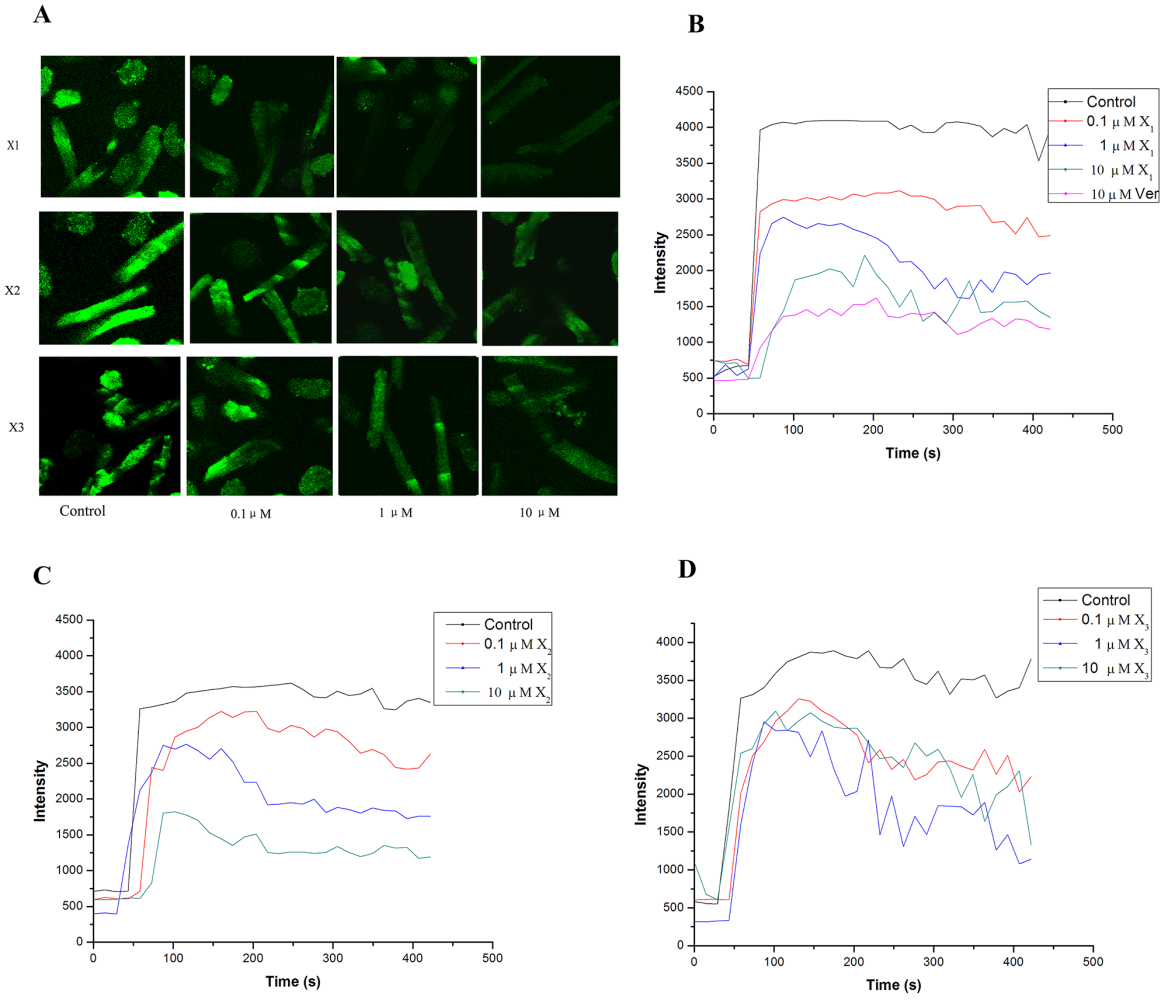
Effect of X_1_, X_2_, X_3_ and the vehicle (0.1% DMSO, control) on KCl (60 mM)-induced changes of Ca^2+^ concentration in rat myocardial cell A: representative variations of Ca^2+^ fluorescence intensities to KCl ; B, C, D: dose-dependent inhibitory effect on the Ca^2+^ fluorescence intensities response to KCl

The effect of X_1_, X_2_ and X_3_ on calcium influx was investigated on rat myocardiocytes. In this experiment, 60 mM KCl was added to induce the myocardial extracellular calcium influx. Ca^2+^ fluorescent intensity increased when KCl was added. The verapamil (10 μM) was used as positive control. It reduced Ca^2+^ fluorescence intensities by 86%, most likely due to block calcium channels and inhibit the KCl-induced extracellular calcium influx [22]. Under the same conditions, use of X_1_, X_2_ and X_3_ (0.1–10 μM) in incubated cells reduced Ca^2+^ fluorescent intensity in a maximum degree by 76.5%, 53.6% and 24.53% respectively (Figure 2). These results demonstrated that the X_1_, X_2_ and X_3_ can inhibit the KCl-induced myocardial intracellular calcium concentration. The inhibitory effects were in the order of X_1_ > X_2_ > X_3_. These results were in consistent with their effects of vasodilation activity.

### Inhibition of L-type calcium current in rat myocardiocytes

**Figure 3:**
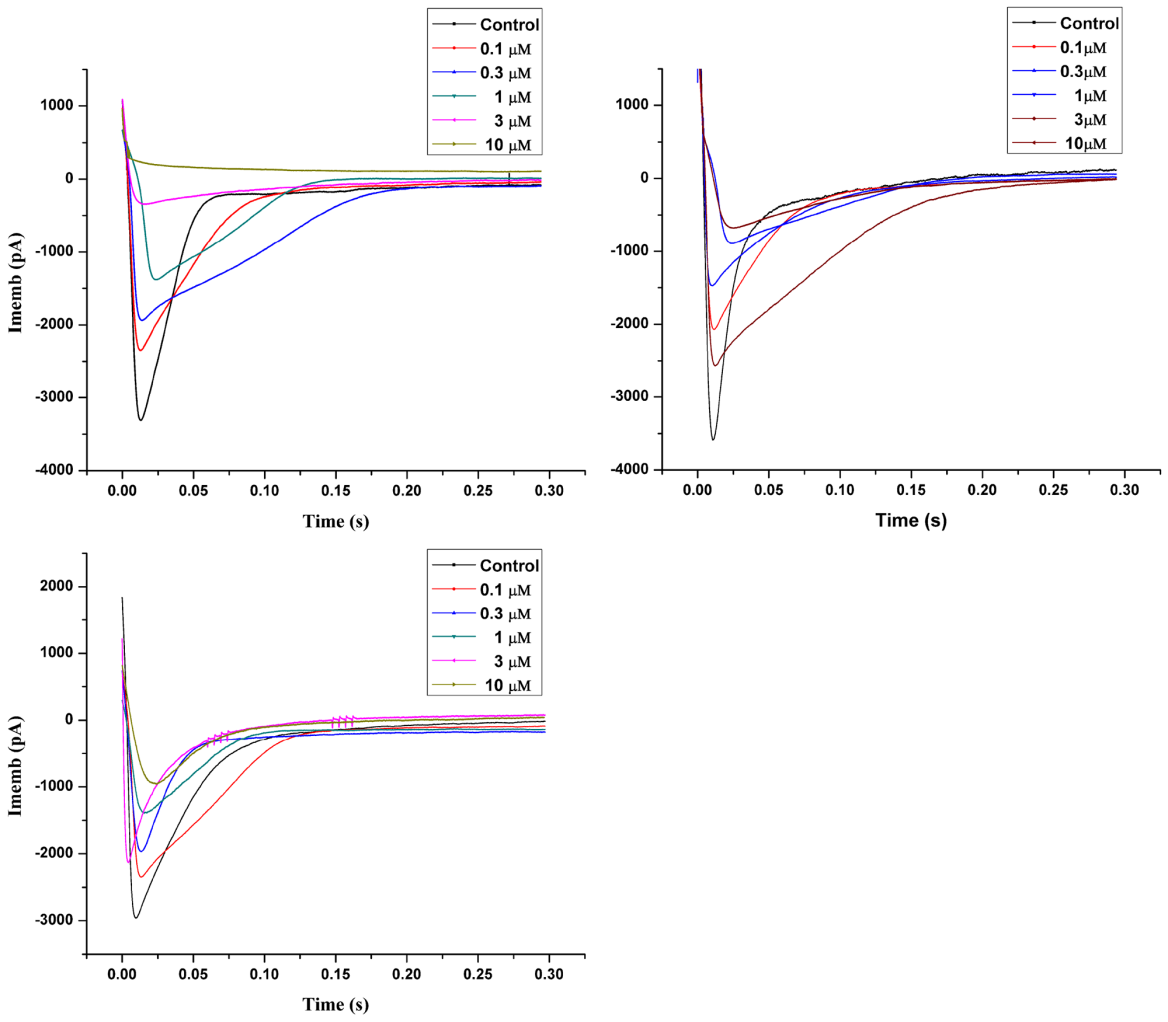
Inhibition of X1, X2 and X3 on L-type calcium current in rat myocardial cell

In the current study, we observed that X_1_, X_2_ and X_3_ (0.1 to 10 μM) reduced the amplitude of the L-type calcium current in rat myocardiocytes (Figure 3). The maximum inhibition rate was 97.2%, 82.8% and 70.2%, and the IC_50_ was 0.472, 0.493 and 0.721 μM respectively for X_1_, X_2_ and X_3_. It was noted that X_1_, X_2_ and X_3_ uniformly blocked the L-type calcium current at different levels of membrane potential in the order of X_1_ > X_2_ > X_3_, but did not change the maximum activation potential and the reversal potential of the calcium channels. These results implicated that X_1_, X_2_ and X_3_ can block the L-type calcium channels in rat ventricular myocytes. This was also in consistent with the result of confocal laser scanning microscopy in which X_1_, X_2_ and X_3_ lowered the concentration of calcium in ventricular myocytes. This result suggested that the underlying mechanism of vasodilation might be related to the characteristics of the L-type calcium channel blockers. To further confirm this, we compared the inhibition of L-type calcium current between X_1_, X_2_, X_3_ and classical calcium antagonists Ver and Nif., The IC_50_ of Ver and Nif were 0.501 uM and 0.524 uM respectively. It was showed that the IC_50_ of X_1_ and X_2_ were less than that of Ver and Nif, and the maximum inhibition rate of X_1_ was same as that of Ver, maximum inhibition rate of X_2_ was almost the same as that of Nif, while maximum inhibition rate of X_3_ was weaker than that of Ver and Nif (Figure S2).

### Comparison of LDH under H/R in H9c2 cell and H9c2 cell (Cacnalc−/−) treated by X_1_, X_2_ and X_3_

Comparing to the control groups, LDH level in the H/R group was significantly increased (*p* < 0.01). When treated with X_1_, X_2_ and X_3_ at the concentration of 1 × 10^−6^ mol/L, LDH decreased (*p* < 0.01) in H9c2 cells, but remained unchanged in Cacnalc−/− H9c2 cell. (Figure S3).

### The computer-fitting molecular docking with L-type channel proteins

**Figure 4:**
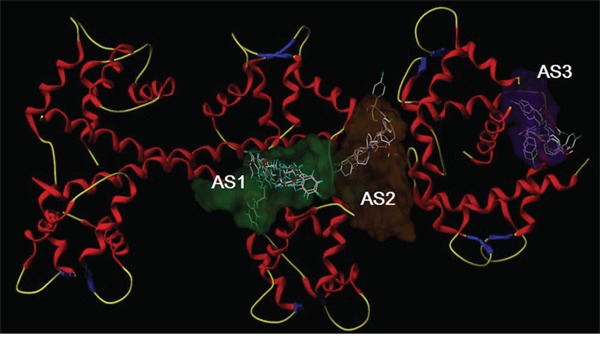
Molecular docking of X1, X2 and X3 in the three putative active sites

**Figure 5:**
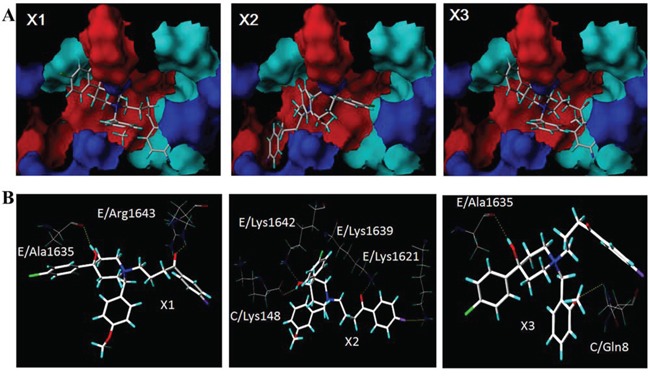
The binding modes of X_1_, X_2_ and X_3_ A. The intermolecular hydrogen bond networks of the three haloperidol derivatives and the Ca^2+^-CaM/CaV1.2 (preIQ-IQ motif) complex B

The automatic detection of Ca^2+^-CaM-CaV1.2 (preIQ-IQ motif) of the L-type calcium channel found the binding sites AS1, AS2 and AS3, as well as their best docking conformation of haloperidol derivatives (Figure 4). The docking scores of each haloperidol derivative were higher at the binding site AS1 than that in the others (Table 2). These findings suggested that AS1 was the most likely binding site of haloperidol derivatives and AS1 was selected for the subsequent binding mode analysis. The mode of binding of each haloperidol derivative to Ca^2+^-CaM-CaV1.2 (preIQ-IQ motif) is shown in Figure 5A. The compounds X_1_ and X_3_ had a similar mode of action. Their A rings interacted with the hydrophobic domain A; their D rings interacted with the hydrophobic domain B. Being differ from X_1_ and X_3_, the A ring and D ring of the compound X_2_ were located in the positively-charged domain and involved in the cation-π interaction. The positively-charged nitrogen of these three compounds had a similar mode of interaction and was in close proximity to the carboxyl group of C-chain Lys148, about 5.5Å in distance, making a strong Coulomb force interaction. In addition, the hydrogen bonding also played an important role in the interaction of the haloperidol derivatives and Ca^2+^-CaM-CaV1.2 (preIQ-IQ motif) as Figure 5B. These results showed that X_1_, X_2_ and X_3_ had a strong interaction with the important complex Ca^2+^-CaM-CaV1.2 (preIQ-IQ motif) of L-type calcium channel. The affinity of the three haloperidol derivatives to Ca^2+^-CaM-CaV1.2(preIQ-IQ motif) was in the order of X_1_ > X_2_ > X_3_.

**Table 2:** The docking scores of three haloperidol derivatives in three putative binding sites

	AS1	AS2	AS3
Compound X_1_	9.0579	8.1259	6.9562
Compound X_2_	8.9876	8.0406	7.2308
Compound X_3_	8.9873	8.7444	7.5680

## DISCUSSION AND CONCLUSIONS

In our previous study, several haloperidol derivatives were synthesized and confirmed to have vasodilation effect. In order to screen new haloperidol derivatives and examine the relationship of their structure and activities, three new ones X_1_, X_2_ and X_3_ were synthesized. Compared to previously synthesized haloperidol derivatives, X_1_, X_2_ and X_3_ showed stronger biological activity, and their water solubility increased.

In the present study, we investigated the side effects of these molecules on the heart. Comparing with classical calcium antagonists, although the potency of X_1_, X_2_ and X_3_ is less than that of verapamil, these novel antagonists neither caused tachycardia nor bradycardia. In contrast, classical calcium antagonists, such as nifedipine activate the sympathetic nervous system and cause tachycardia, while verapamil and diltiazem cause bradycardia and conduction block. Therefore classical calcium channel blockers were not recommended for patients with heart failure, sinus node or atrioventricular node dysfunction. Since X_1_, X_2_ and X_3_ had little side effect on the heart and did not affect the diastolic function, they could be widely used in clinical practice.

The model of KCl-induced aortic ring contraction is the classic method *in vitro* for screening vasodilation drugs. The present study showed that X_1_, X_2_ and X_3_ had vasodilation activity in a dose-dependent manner in the KCl-induced aortic ring contraction model. Studies have demonstrated that the contraction of vascular smooth muscle is initiated by increased intracellular calcium level [20–22], which may be achieved by two ways: extracellular Ca^2+^ influx from voltage-depend calcium channel (VDCCs) evoked by depolarization with high potassium concentration and intracellular Ca^2+^ release from intracellular storage [6, 23, 24]. It is known that vasoconstriction induced by KCl (60 mM) was due to the influx of extracellular Ca^2+^ [10]. The X_1_, X_2_ and X_3_ caused the same vasoconstriction in both endothelium-intact and endothelium-free rat aortic rings (data no shown). It indicated that this kind of vasoconstriction was not related to vasodilation factor NO of vascular endothelial cells [12, 13], but related to the influx of extracellular Ca^2+^ which is induced by KCl (60 mM).

In order to study the effects of haloperidol derivatives on extracellular calcium influx, the laser scanning confocal microscopy (LSCM) was used to observe the changes of intracellular calcium concentration after extracellular calcium influx. The results of the effect on KCl-dependent Ca^2+^ signaling indicated that X_1_, X_2_ and X_3_ decreased the influx of extracellular Ca^2+^ caused by depolarization in myocardial cell.

The extracellular calcium influx through the voltage-dependent L-type calcium channel plays the most important role in the regulation of a stable level of free calcium in cells [21]. There are 6 types of calcium channels, i.e. L, T, N, P, R, and Q types. In myocardiocytes, L- and T-types are the most common ones [23]. L-type calcium channel is the main channel responsible for extracellular calcium influx and cell contraction [24]. L-type channel is particularly sensitive to calcium channel blockers [25]. The whole cell patch clamp recording is usually used to observe the electric current of a certain type of ion channels carried on the membrane. This technique is supposed to be the most direct means to study the effects of drugs on the activity of ion channel [26]. In the current study, we investigated the L-type calcium current by setting the holding potential to - 40 mV to exclude the impact of T-type calcium current and sodium current. Tetraethyl ammonium was added in the extracellular fluid and 4-AP in the pipette solution to block the impacts of multiple types of potassium current. The recorded current was blocked by the positive control drug verapamil [27]. Our results demonstrated that the underlying mechanism of vasodilation of X1, X2 and X3 was related to the characteristics of the L-type calcium channel blockers.

We used H9c2 cell and calcium channel deleted cells, the H9c2 cell (Cacnalc−/−) and performed the hypoxia/reoxygenation (H/R) experiments. We observed the changes of LDH which is a classic indicator of myocardial cell injury under H/R. The result showed that LDH decreased in in H9c2 cell, and remained unchanged in Cacnalc−/− H9c2 cells upon treatment with X_1_, X_2_ and X_3_. This result indicated that the effect of X_1_, X_2_ and X_3_ against injury under H/R was attributed to the blocking of L-type calcium channel.

In order to analyze the mechanism for the inhibition of L-type calcium channels by haloperidol derivatives, the important regulatory complex Ca^2+^-CaM-CaV1.2 (preIQ-IQ motif) of the L-type calcium channel was selected as the docking receptor, followed by molecular docking analysis. The results showed that X_1_, X_2_, X_3_ had a strong interaction with the important complex Ca^2+^-CaM-CaV1.2 (preIQ-IQ motif) of L-type calcium channel. Ca^2+^-CaM and CaV1.2 interaction is a dual-direction regulation on L-type calcium channel, including Ca^2+^-dependent facilitation (CDF) and Ca^2+^-dependent inactivation (CDI). Past studies reported that the CaV1.2 preIQ interacting with Ca^2+^-CaM involves in the regulation of CDI [29–31]. The docking results showed that the haloperidol derivatives stabilized the Ca^2+^-CaM/preIQ complexes by hydrophobic effect and hydrogen bonds, so that Ca^2+^-CaM/preIQ complexes sustained the function of CDI, thereby inhibiting extracellular calcium influx. The docking results showed that the affinity of the three haloperidol derivatives to Ca^2+^-CaM-CaV1.2(preIQ-IQ motif) was in the order of X_1_ > X_2_ > X_3_. Accordingly, the effects of these three haloperidol derivatives on L-type calcium channels were X_1_ > X_2_ > X_3_.

In summary, the *N*-methoxy-benzyl haloperidol quaternary ammonium derivatives are a structurally novel class of calcium antagonists. They reduced the concentration of intracellular calcium by inhibiting extracellular calcium influx. The SAR of result showed that the activities of X_1_, X_2_ and X_3_ were closely related to the interactions between the compounds and the L-type calcium channel proteins. The findings of this study provide a new idea for developing a structurally novel class of calcium antagonists with less cardiac side effects.

## MATERIALS AND METHODS

### Synthesis of para-, meta- and ortho-substituted N-methoxy-benzyl haloperidol derivatives (X_1_, X_2_, X_3_)

The heating reflux equipment was from Dafeng glassware Co., Ltd, China. It consisted of a heater, an evaporator, a condenser and a cooler. Haloperidol (10 mM) was dissolved in the corresponding benzyl chloride (4 ml). The mixture was heated in an oil bath for 24 h to maintain reflux and then cooled to room temperature. The filtered solid was recrystallized from an ethanol-water mixed solvent to a crystalline product. 1H NMR spectra were recorded on a Bruker AM-400 spectrometer (400 MHz) (Bruker, Switzerland). Yields are within 90% of the theoretical values. The purity of all compounds were determined to be about 95% using analytical reversed-phase high performance liquid chromatography (RP-HPLC) (Waters 600 / 2478, USA) and detected with MS for details. No UV-active impurities were observed at 245 nm with RP-HPLC-UV.

### The effect of haloperidol derivatives on rat hemodynamics

SD rats of either sex weighing between 220–250 g were anesthetized with intravenous pentobarbital sodium (30 mg/kg). A cannula were linked to a pressure transducer (PT14M2, Fudan university, China) filled with 40 IU/ml of sodium heparin. It was inserted into the femoral artery to monitor mean arterial pressure and inserted into the left ventricle through right common carotid artery to measure the left ventricular pressure. The changes of hemodynamics, including left ventricular systolic pressure (LVSP), left ventricular end-diastolic pressure (LVEDP), ± dP/dtmax and heart rate (HR) were recorded with MS302 recording-and-analyzing system software (Guangdong Medicine College, China) at baseline and 3 min after administration of drugs.

### Vasodilation activity on rat aortic ring

The vasodilation activity of compounds was evaluated in isolated rat thoracic aortic rings according to the methods of Greenwood and Polster et al [9, 10]. The aortic rings were allowed to equilibrate in Krebs bicarbonate solution ((PSS), containing118.0 mM NaCl, 4.7 mM KCl, 1.2 mM KH_2_PO_4_, 1.2 mM MgSO_4_.7H_2_O, 5.0 mM glucose, 25.0 mM NaHCO_3_, and 2.5 mM CaCl_2_.7H_2_O at 37°C and were gassed with 95% O_2_ and 5% CO_2_ for 90 min at a testing tension. KCl (60 mM) was used to depolarize the aortic rings. When the amplitude of the contraction reached a plateau, compounds X_1_, X_2_, X_3_ were added. Cumulative concentration-response curves to all compounds were determined in the absence and presence of endothelium. Using tips of forceps, the endothelium was mechanically removed from some rings by gently rubbing the lumen, if absence of endothelium is needed. Acetylcholine was used to confirm the removal of endothelium from the aortic rings [11].

### Inhibition of KCl-induced rat myocardial extracellular calcium influx

Fluorescence intensity of Ca^2+^ in myocardial cell was determined by laser scanning confocal microscopy (ACAS/ULTIMA312, Meridian Instruments) as previously described [12]. Rat myocardiocytes were grown on 24-mm glass coverslips in 6-well plates in DMEM containing 10% FBS. After 24 h, the cells were incubated with Fluo 3-AM (20 μM) for 60 min in the dark at 37°C. Then, the cells were washed with HBSS three times to remove extracellular Fluo 3-AM. HBSS (HEPES-buffered physiological saline solution) contains 130 mM NaCl, 2.5 mM KCl, 1.2 mM MgCl_2_, 10 mM HEPES, 10 mM glucose, and 2 mM CaCl_2_, pH 7.4 (adjusted with NaOH). The Fluo-3 dye was excited with a 488-nm wavelength argon laser beam, and the emission fluorescence was monitored at 530 nm. KCl was added into the cultured cells to induce the influx of extracellular Ca^2+^ in HBSS and changes in fluorescence were recorded. Myocardiocytes were incubated for 30 min with 0.1, 1, and 10 μM compounds before adding KCl. Effect of compounds on KCl-induced changes in fluorescence was investigated.

### Inhibition of L-type calcium current in rat Myocardiocytes

The patch-clamp technique in the perforated-attached configuration [13] was used to study Inward barium (*I*_Ba_) currents in single cells. Records were made using a Biologic RK-300 amplifier, filtered (23 dB, 5-pole Tchebicheff filter) at 1 kHz and sampled at 5 kHz. Currents were recorded from holding potential 240 mV during linear voltage ramps from 240 to +60 mV, with 10 mV increments. Patch pipettes filled with the pipette solution containing 125 mM NaCl, 10.8 mM BaCl_2_, 1.0 mM MgCl_2_, 5.4 mM CsCl, 10 mM glucose, and 10 mM Hepes, pH = 7.4. The electrode resistance ranged from 2–5 MV. Drugs were applied in the bath solution. The final concentration of ethanol and the diluent was 0.1% (v/v). The pClamp 8.1 software (Axon Instruments) was used for sampling and data analysis.

### Measurements of Lactate dehydrogenase (LDH) in myocardiocytes culture medium

Myocardiocytes H9c2 cell and the one which the L-type calcium channel gene was knock-out, the H9c2 (Cacnalc−/−) were incubated separately in culture plates. When cell confluence reached 85%, the cells were incubated for another 12 hours in DMEM medium with 0.5% FBS. Then cells were randomly divided into 6 groups, namely the control group, H/R group, DMSO group, H/R+X_1_(1 × 10^−6^ mol/L) group, H/R+X_2_ (1 × 10^−6^ mol/L) group and H/R+X_3_ (1 × 10^−6^ mol/L) group. The hypoxic DMEM culture medium and reoxygenation medium were used in H/R process. The final volume was 500 uL. The compounds (X_1_, X_2_, X_3_) were administered continuously during the hypoxia/reoxygenation process. Finally, cell supernatant was collected and LDH was measured by colorimetric method according to the Kit instruction by manufacturer (Nanjin Jiancheng Lit. China).

### X-ray diffraction of single crystal for studying spatial structure

Crystal spatial structure data was collected by Bruker SMART 1000 CCD diffractometer and absorption was corrected by multi-scan with Tmin = 0.938 and Tmax = 0.947. SAINT-Plus (Bruker, 2003) was used for data reduction and for cell refinement [14]. SHELXS97 program(s) was used to analyze the structures [15].

### The computer-fitting molecular docking with L-type channel proteins

#### Ligands and receptors

X-ray crystal structures of these three haloperidol derivatives were used as initial structures for molecular structure optimization. The optimization of the molecules was carried out using a Powell method with a convergence limit of 0.05 kcal/mol/Å. The Tripos force field and Gasteiger–Hückel partial charges were applied in the calculations. Then a molecular database of haloperidol derivatives was set up. The crystal structure of Ca2+-CaM/CaV1.2 (preIQ-IQ motif) complex was downloaded from the Protein Database Bank (PDB ID: 3G43). Removing chain A and waters, the complex was prepared by adding terminators, hydrogen, charges and fixing side chains using the Biopolymer Structure Preparation Tool in SYBYL X 1.2. The structure of complex was optimized using the Powell method and with a convergence limit of 0.05 kcal/mol/Å. Amber FF99 and Amber partial charges were applied for calculations.

#### Molecular docking

Molecular docking was performed using the Surflex-Dock algorithm, which is available in the SYBYL X 1.2. Surflex-Dock is a fast, flexible docking method that employs an idealized active site ligand (called protomol) as a target for generating putative poses of molecules. These putative poses are scored using the Hammerhead scoring function. The scoring function contains the hydrophobic, polar, repulsive, entropic, solvation and crash terms. The scores are expressed in units of –log(Kd) to represent binding affinities. The binding sites of haloperidol derivatives were detected automatically by Surflex-Dock. After generation of protomols, the Surflex-Dock Geom X mode was applied to perform a high precision docking, leveled all parameters in their default values. The docked poses with the highest docking scores were selected for further analysis of binding modes.

## SUPPLEMENTARY TABLES AND FIGURES



## Abbreviations

X1: quaternary ammonium salt derivatives of haloperidol with N-p-methoxybenzyl
X2: quaternary ammonium salt derivatives of haloperidol with N-m-methoxybenzyl
X3: quaternary ammonium salt derivatives of haloperidol with N-o-methoxybenzyl groups
LVSP: left ventricular systolic pressure
LVEDP: left ventricular end-diastolic pressure
HR: heart rate
LSCM: laser scanning confocal microscopy
VDCCs: voltage-depend calcium channel
CDF: Ca^2+^-dependent facilitation
CDI: Ca2+-dependent inactivation
