# Supplementary material for: Characterization and bioactivity of novel calcium antagonists - N-methoxy-benzyl haloperidol quaternary ammonium salt
Source: Oncotarget. 2015 Oct 19;6(41):43759–69. doi: 10.18632/oncotarget.6086 (PMC4791264; doi:10.18632/oncotarget.6086)
Supplement: Supplementary file 1 [file oncotarget-06-43759-s001.pdf]

SUPPLEMENTARY TABLES AND FIGURES

Supplementary Table S1: Chemical and physical characteristics of compounds

Compound	R	Yield, %	Mp, °C	Molecular formula
**X3**	2-OCH_3	95%	228~230	$C_{29}H_{32}NCl_2FO_3$
**X2**	3-OCH_3	92%	241~243	$C_{29}H_{32}NCl_2FO_3$
**X1**	4-OCH_3	94%	208~211	$C_{29}H_{32}NCl_2FO_3$

Supplementary Table S2: The IR data, HNMR data, EI-MS data of compounds

Compound	IR/KBr, cm^{-1}	^{1}HNMR/300 MHz, $CDCl_3$-d_1/DMSO-d_6	EI-MS (m/z)
**X3**	1684(C = O)	2.19-2.23(m,2H,COCCH2C);2.33-2.40(t,4H,N(CCH2)2C);3.26-3.29(t,2H,COCH2);3.41-3.51(m,4H,N(CH2)2);3.54-3.60(t,2H,COCCCH2);3.88(s,3H,OCH3);4.63(s,2H,N-CH2-Ar);5.55(s,H,OH);7.09-7.13(d,H,Ar-H);7.21-7.23(d,H,Ar-H) 7.39-7.45(m,4H,Ar-H); 7.54-7.61 (m,4H,Ar-H); 8.10-8.13 (m,2H, Ar-H);	498(M+2)<br>496(M +)
**X2**	1684(C = O)	2.14-2.24(m,2H,COCCH2C);2.35-2.41(t,4H,N(CCH2)2C);3.29-3.32(t,2H,COCH2);3.43-3.54(m,4H,N(CH2)2);3.59-3.69(t,2H,COC CCH2);3.82(s,3H,OCH3);4.69(s,2H,N-CH2-Ar);5.62(s,H,OH);7.11-7.14(d,H,Ar-H);7.22-7.24(d,H,Ar-H) 7.28(s,H,Ar-H);7.39-7.48(m,5H,Ar-H); 7.59-7.61 (d,2H,Ar-H); 8.09-8.13 (m,2H, Ar-H);	498(M+2)<br>496(M +)
**X1**	1684(C = O)	2.26-2.33(m,2H,COCCH2C);2.43-2.51(t,4H,N(CCH2)2C);3.25-3.28(t,2H,COCH2);3.34-3.49(m,4H,N(CH2)2);3.83(s,3H,OCH3);4.33-4.39(t,2H,COCCCH2);4.68(s,2H,N-CH2-Ar);5.77(s,H,OH);6.95-6.97(d,2H,Ar-H);7.15-7.19(d,2H,Ar-H);7.32-7.34(d,2H, Ar-H)7.50-7.53(d,2H,Ar-H); 7.66-7.67 (d,2H,Ar-H); 8.01-8.04 (m,2H, Ar-H)	498(M+2)<br>496(M +)

Supplementary Table S3: Crystal data and structure refinement for compounds

Compound reference	Compound X_1	Compound X_2	Compound X_3
Chemical formula	2*C29 H32 Cl2 F N O3	C29 H32 Cl2 F N O3	C29 H32 Cl2 F N O3
Crystal system	Triclinic	Triclinic	Triclinic
a/Å	10.2730(10)	9.300(7)	9.6366(15)
b/Å	17.6515(17)	10.218(7)	10.8760(16)
c/Å	18.0048(17)	14.627(11)	13.442(2)
α/°	103.839(2)	83.658(13)	103.507(2)
β/°	100.303(2)	76.706(13)	100.992(2)
γ/°	94.492(2)	88.722(13)	99.171(2)
Unit cell volume/$Å^3$	3093.83	1344.43	1313.68
Space group	P -1	P -1	P -1
No. of formula units per unit cell, *Z*	2	2	2
Final R_1 values (all data)	0.06	0.05	0.05

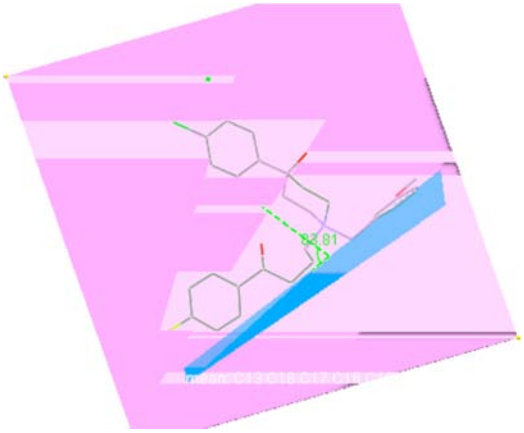

Supplementary Figure S1: Ring plane dihedral angle in compounds

Supplementary Figure S1A: A and C ring plane dihedral angle in X_3: 83.81(3).

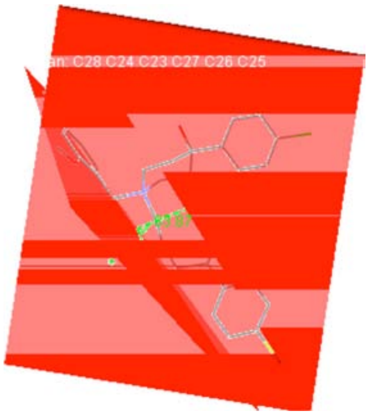

Supplementary Figure S1B: A and C ring plane dihedral angle in X_2: 83.87(2).

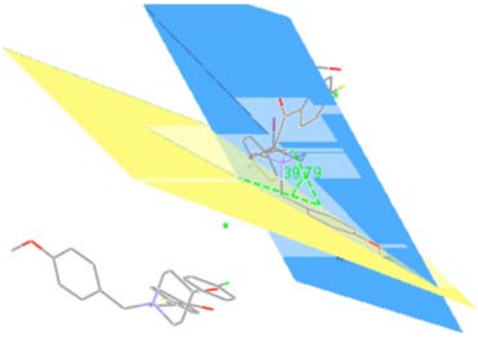

Supplementary Figure S1C: A and C ring plane dihedral angle in X_1: 39.70(2).

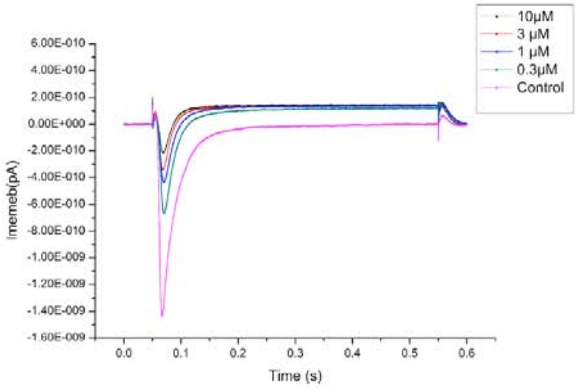

Nif

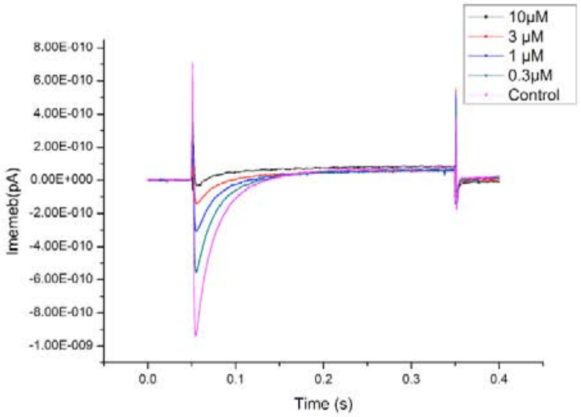

Ver

Supplementary Figure S2: Comparison of the inhibition of L-type calcium current between X_1, X_2, X_3 and classical calcium antagonists Ver and Nif.

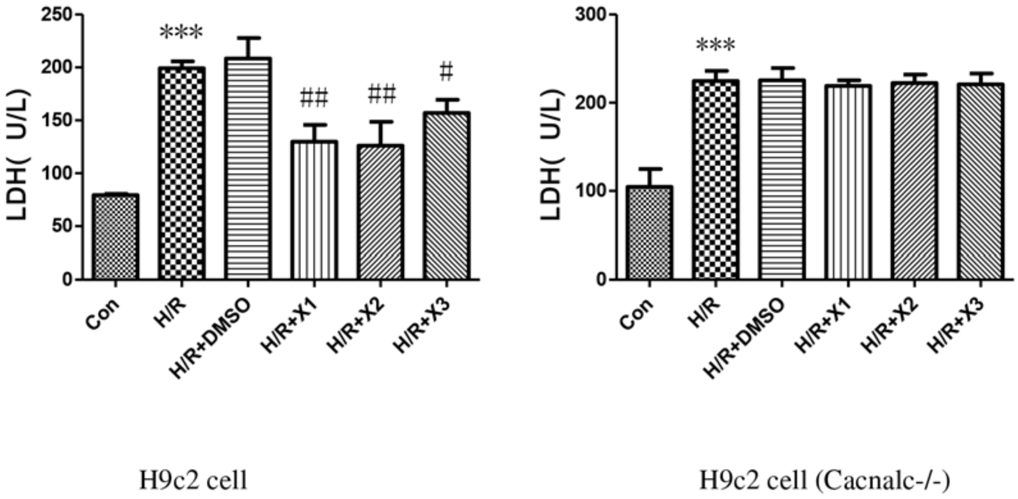

Supplementary Figure S3: LDH level in H9c2 cell and the H9c2 cell (Cacnalc-/-) with hypoxia/reoxygenation (H/R) experiments.
